# Effect of fly ash and curing temperature on the properties of magnesium phosphate repair mortar

**DOI:** 10.1038/s41598-024-66581-1

**Published:** 2024-07-05

**Authors:** Junxia Liu, Jingyu Zhang, Anbang Li, Xiaomin Xia, Junpeng Chen

**Affiliations:** School of Architectural Engineering, Zhongyuan University of Technology, Zhengzhou, 450007 Henan China

**Keywords:** Curing temperature, Fly ash, Interfacial bonding strength, Magnesium phosphate repair mortar, Mechanical properties, Water resistance, Environmental sciences, Materials science

## Abstract

This article is aimed at discussing the combined effect of mineral admixture and servicing temperature, especially in cold environment, on the properties of magnesium phosphate repair mortar (MPM). The influence mechanism of fly ash content on the microstructure and performance of MPM were firstly investigated, and then the evolution rules in properties of fly ash modified MPM cured at − 20 °C, 0 °C, 20 °C and 40 °C were further revealed. The results show that the incorporation of fly ash has no significant effect on the setting time and fluidity of MPM. When MPM is modified with 10 wt% and 15 wt% fly ash, its mechanical properties, adhesive strength, water resistance, and volume stability are effectively improved. Fly ash reduces the crystallinity and continuity of struvite enriched in hardened MPM, and its particles are embedded among struvite and unreacted MgO. The compressive strength of MPM-10 cured for various ages increases with the elevating of curing temperature, while the flexural strength, interfacial bonding strength, strength retention and linear shrinkage exhibits the opposite laws. When cured at 0 °C and − 20 °C, MPM-10 still has good early strength, water resistance and interfacial bonding properties, which indicates that MPM-10 provides with an ability of emergency repair of cracked components served in cold environments.

## Introduction

Magnesium phosphate repair mortar (MPM) is a new type of repair material prepared from acid phosphate, dead-burned magnesium oxide (MgO), fine aggregates, chemical additives and mineral admixtures. MPM is characterized by fast-hardening and early-strengthening, low-temperature condensation, and has good volume stability, interfacial bond strength and durability^1,2^. Based on this, MPM is commonly used in the fields of structural repair and cold environment construction such as airport runways, highways, bridges and municipal trunk roads, but its promotion and application are limited due to its high hydration temperature, poor water resistance and high cost^3–5^.

Above mentioned problems can be alleviated by mineral admixtures, such as silica fume, blast furnace slag, metakaolin, red mud and fly ash, by means of their active, morphological and filling effects^6–8^. Silica fume partially replacing potassium dihydrogen phosphate and dead-burned MgO, could reduce the hydration rate and hydration heat of magnesium phosphate cement (MPC)^9^. Tan et al.^10^ found that the strength retention of MPC was dramatically improved at 60 days of immersion in freshwater or simulated seawater when the slag content was 30 wt% or higher. Liu et al.^11^ indicated that red mud effectively reduced the exothermic rate of MPM, and improved the mechanical properties and water resistance at each curing age with a replacement rate of 10–20 wt%. Qin et al.^12^ demonstrated that metakaolin optimized the microstructure, water resistance, and dimensional stability of MPM when its content increased to 30 wt%. Mineral admixtures refine the pore structure of hardened MPC, resulting in some improvement in its water resistance, mechanical properties, and economy.

Fly ash is the most widely used mineral admixture, and its application in MPC and MPM has also received extensive attention^13^. Dong et al.^14^ found that a substitution rate of 10 wt% fly ash not only reduced the hardening rate and viscosity of fresh MPC, but also improved its compressive strength, water resistance, and dimensional stability. Fly ash promoted the formation of hydration products of MPC, and optimized the mechanical properties, compactness and water resistance at 7 days and 28 days^15–17^. Experimental studies of Li et al.^18^ showed that MPM composite modified with 10 wt% fly ash and 20 wt% alumina cement had a good bonding strength and lower drying shrinkage rate, reducing the risk of spalling of repair materials. An appropriate content of fly ash is beneficial to the mechanical properties of MPC and its repair mortar, achieving more significant effects on improving its volumetric stability.

Compared with traditional cement-based repair materials, MPC offers the property of condensation and hardening in negative temperatures, which provides a new technical approach for the rapid repair of in-service structures under severe cold environments. The essence of MPC hydration is acid–base neutralization reaction, although its rate decreases in various degrees under a negative temperature environment, MPC still has suitable early mechanical properties^19,20^. Experimental studies by Luo et al.^21^ showed that the setting time of MPC extended from 10 min to more than 30 min under negative temperature conditions. Jia et al.^22^ indicated that light-burned MgO quickly reacts with acid phosphate and releases a large amount of hydration heat, the generated struvite further reduces the freezing point of the water, which ensured the normal hydration of MPC. When the content of light-burned MgO was 8 wt%, the compressive strength of MPC reached 32.0 MPa curing at – 20 °C for 2 h. Therefore, the effect of cold conditions on the set-hardening characteristics and mechanical properties of MPC and its repair mortar in is worth system explored if without changing the composition and characters of raw materials.

MPC and its mortar have the characteristics of condensation and hardening in negative temperature environment, which undoubtedly expands their application range. Based on the previous research^23^, in this paper, the modified MPM was prepared by partially replacing ammonium dihydrogen phosphate (NH_4_H_2_PO_4_) and dead-burned MgO with fly ash, and the influence mechanism of the fly ash replacement rate on the physical and mechanical properties, such as setting time, fluidity, mechanical properties, interfacial bonding strength, water resistance and volume stability, and microstructure of the MPM was investigated. Taking optimal fly ash modified MPM as the research object, the variations of its physical and mechanical properties with curing temperature, such as − 20 °C, 0 °C, 20 °C, and 40 °C, were further revealed. The research reveals the difference in macro-performance of MPM at negative and normal temperature, and provides basic data for its application in emergency repair projects under severe cold environment.

## Experimental materials and methods

### Materials

**Figure 1:**
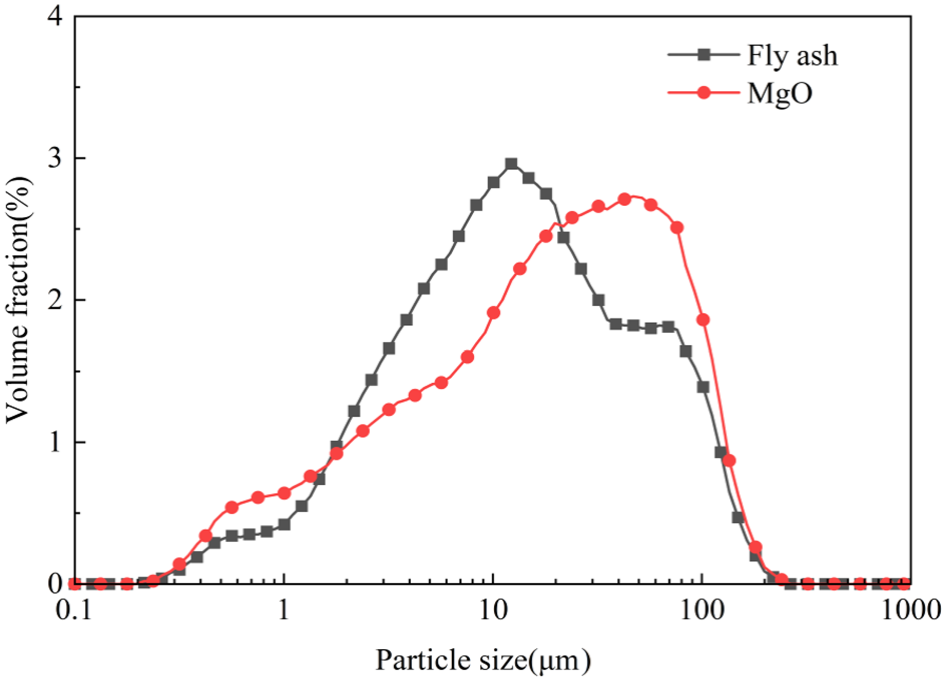
Particle size distribution of calcined magnesia and fly ash.

The dead-burned MgO was calcined from magnesite at 1780 °C, its chemical composition is listed in Table 1, its particle distribution is shown in Fig. 1, and its specific surface area and average particle size were 764.0 m^2^/kg and 28 μm, respectively. NH_4_H_2_PO_4_ was industrial grade chemical reagent, its purity was greater than 90 wt%. Sodium tetraborate, borax, was an analytical pure reagent, its purity was greater than 99.5 wt%. The chemical composition of fly ash is listed in Table 1, and its particle distribution is also shown in Fig. 1. The quartz sand contented 99.5 wt% SiO_2_, and its particle size range was 0.45–0.71 mm, that was 26–40 mesh. Industrial sugar was used as retarder with a sucrose content of 98.0 wt%.

**Table 1:** Chemical composition of dead-burnt magnesia oxide and fly ash (wt%).

Raw materials	MgO	SiO_2_	Al_2_O_3_	Fe_2_O_3_	CaO	K_2_O	SO_3_	TiO_2_	P_2_O_5_	LOI
Dead-burned MgO	91.46	2.03	1.02	1.27	1.75	0.04	0.20	0.04	0.13	2.06
Fly ash	0.57	54.54	31.18	3.42	3.06	1.53	0.21	0.94	0.18	4.18

### Mixtures of MPM

The mixtures of MPM are shown in Table 2. In which, M/P is the mass ratio of MgO to NH_4_H_2_PO_4_, W/C is mass ratio of water to cementitious materials, which was the total mass of dead-burned MgO, NH_4_H_2_PO_4_ and fly ash. B/M is for the mass ratio of borax to MgO, S/C represents the ratio of quartz sand to cementitious materials, and IS/B is the mass ratio of industrial sugar to borax. The replacement rates of fly ash were 0 wt%, 5 wt%, 10 wt%, 15 wt%, 20 wt%, 25 wt% and 30 wt% of cementitious materials, respectively, which were expressed as MPM-0, MPM-5, MPM-10, MPM-15, MPM-20, MPM-25 and MPM-30.

**Table 2:** Mixtures of MPM.

Mixture ID	M + P (wt%)	Fly ash (wt%)	M/P	W/C	B/M	S/C	IS/B
MPM-0	100	0	2/1	0.20	0.12	1	0.03
MPM-5	95	5	2/1	0.20	0.12	1	0.03
MPM-10	90	10	2/1	0.20	0.12	1	0.03
MPM-15	85	15	2/1	0.20	0.12	1	0.03
MPM-20	80	20	2/1	0.20	0.12	1	0.03
MPM-25	75	25	2/1	0.20	0.12	1	0.03
MPM-30	70	30	2/1	0.20	0.12	1	0.03

### Preparation methods

In order to make the test conditions as close as possible to the set temperature environment, the pre-treatment mentioned in this paper refers to the treatment of the raw material before the MPM mixing. Firstly, dead-burnt MgO, NH_4_H_2_PO_4_, fly ash, borax and industrial white sugar were weighed according to mixtures of MPM-10 listed in Table 2, and packaged individually in sealed bags. Then, the packaged raw materials were placed in a curing box set with designed temperature, which was 40 °C, 20 °C, 0 °C, and -20 °C, and pretreatment time was no less than 24 h.

Firstly, the pretreated raw materials were slowly stirred for 60 s, and then slowly mixed and stirred for 30 s after adding water, subsequently, quickly mixed and stirred for 60 s. Then, the mixture was poured into the three-connected mold of 40 mm × 40 mm × 160 mm for vibration molding. The humidity curing at 20 °C and 40 °C was set 55 ± 1%, and was no set curing at − 20 °C and 0 °C, the corresponding performance was tested at the specified age.

### Test methods of physical and mechanical properties

#### Setting time and fluidity

**Figure 2:**
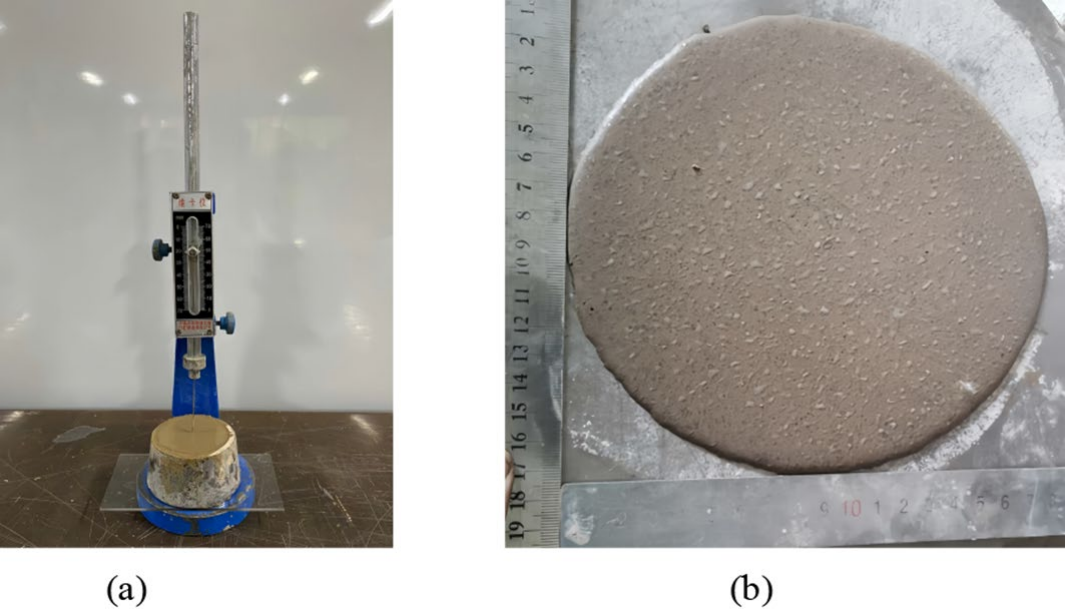
Setting time and fluidity testing of MPM. (**a**) Setting time, (**b**) Fluidity.

The hydration rate of MPM is fast, and the interval between initial and final setting time is very short. Therefore, the initial setting time of MPM obtained by Vicat method was used as the final setting time in this paper. According to Chinese standard GB/T 2419-2005 “The method for determination of fluidity of cement mortar”, the fluidity of MPM at different curing temperatures was tested at 20 ± 2 °C. The testing images of setting time and fluidity are shown in Fig. 2.

#### Mechanical properties

According to Chinese standard JC/T 2537-2019 “Magnesium phosphate repairing mortar”, the compressive and flexural strength of MPM at 1.5 h, 3 days and 28 days at different curing temperatures were tested.

#### Interfacial bonding strength

**Figure 3:**
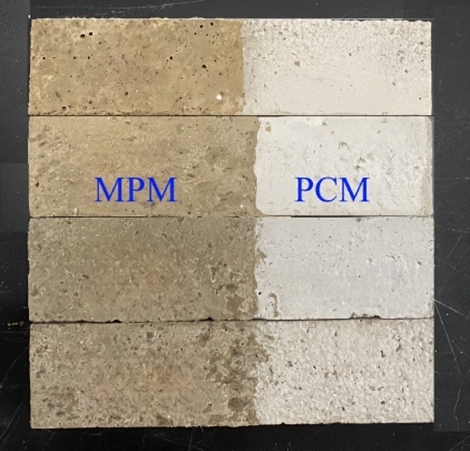
MPM specimens for testing interfacial bonding strength.

Interfacial bonding strength of MPM and old Portland cement mortar (PCM) was measured indirectly by flexural strength, its specimens are showed in Fig. 3. The Portland cement mortar (40 mm × 40 mm × 160 mm) was made according to Chinese standard JC/T 2381-2016, and was divided into two sections of almost uniform sections after cured for 28 days under standard curing conditions. Place half of the Portland cement mortar block into the mold and place the break section in the middle of each mold. Then, the MPM mixture is poured into the remaining space and vibrated to form. Finally, the demolded specimens were placed in a curing box. According to the above standards and setting the curing temperature, the sample was air-cured for 1.5 h and 3 days, the immersed water sample was air-cured for 7 days, and then water-cured for 7 days.

#### Water resistance

The water resistance of MPM was characterized by the compressive strength retention coefficient (${w}_{n}$), which was tested according to Chinese standard GB/T 50082-2009 “Standard for test methods for long-term performance and durability of ordinary concrete”. After 3 days of air curing, the specimens were poured into water and cured 28 days. The specimens with different curing temperatures were placed in the corresponding curing water tank, in which the curing water remained liquid at − 20 °C through antifreeze. The calculation formula of ${w}_{n}$ was as follows:

1 $${w}_{n}=\frac{{f}_{\text{cn}}}{{f}_{c}},$$

where ${w}_{n}$ is the compressive strength retention coefficient. *f*_cn_ is the average compressive strength of MPM cured in water for 28 days, MPa. *f*_c_ is the average compressive strength of MPM air-cured 28 days, MPa.

#### Dry shrinkage

**Figure 4:**
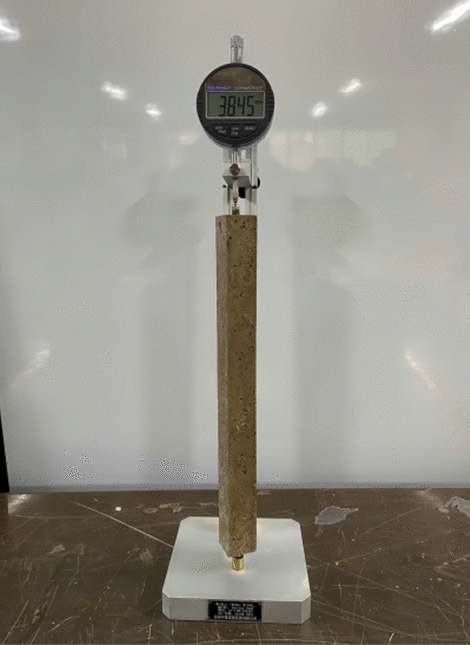
MPM dry shrinkage testing.

The MPM specimens for drying shrinkage were prepared according to Chinese standard JC/T 603-2004 “Cement Mortar Dry Shrinkage Test Method”. After demolding, the specimens were immersed in liquid water at − 20 °C, 0 °C, 20 °C and 40 °C for 3 days, respectively. And then, the specimens were cured in air curing environment at corresponding temperature till the given testing age. The length of the MPM specimens were tested by the comparator with an accuracy of ± 1–± 1.5 μm/200 mm. The testing images of drying shrinkage are shown in Fig. 4.

### Scanning electron microscopy (SEM) and X-ray diffraction (XRD) analysis methods

In order to avoid the interference of quartz sand on EDS element analysis of hardened MPM, the SEM samples were net slurry, which cementitious materials composition and W/C were the same as the corresponding MPM. After curing 28 days, the samples were terminated hydration by soaking in anhydrous ethanol for 7 days. Before the SEM testing, the MPM samples were placed in a vacuum drying oven at 60 °C to constant weight, and then sprayed with carbon to improve their electrical conductivity. The samples were subjected to a Quanta 250 FEG field emission SEM under the conditions of 5 kV accelerating voltage and 3 spot sizes, and their microstructure and morphology of hydration products were observed. At the same time, the elemental composition of the relevant fields was analyzed by flat-scan energy dispersive spectrometry (EDS).

XRD samples were ground and passed through a 75 μm square mesh sieve before XRD analysis. The results were recorded with a step size of 0.02°/min in the range of 10°–65° using a D8 ADVANCE operating under Cu-Kα radiation of 15 mA/40 kV. MDI jade 6 software was used to analyze the mineral composition of hydration products.

## Results and discussion

### Effect of fly ash content on the physical and mechanical properties of MPM cured at 20 °C

#### Setting time and fluidity of MPM with different fly ash content

**Figure 5:**
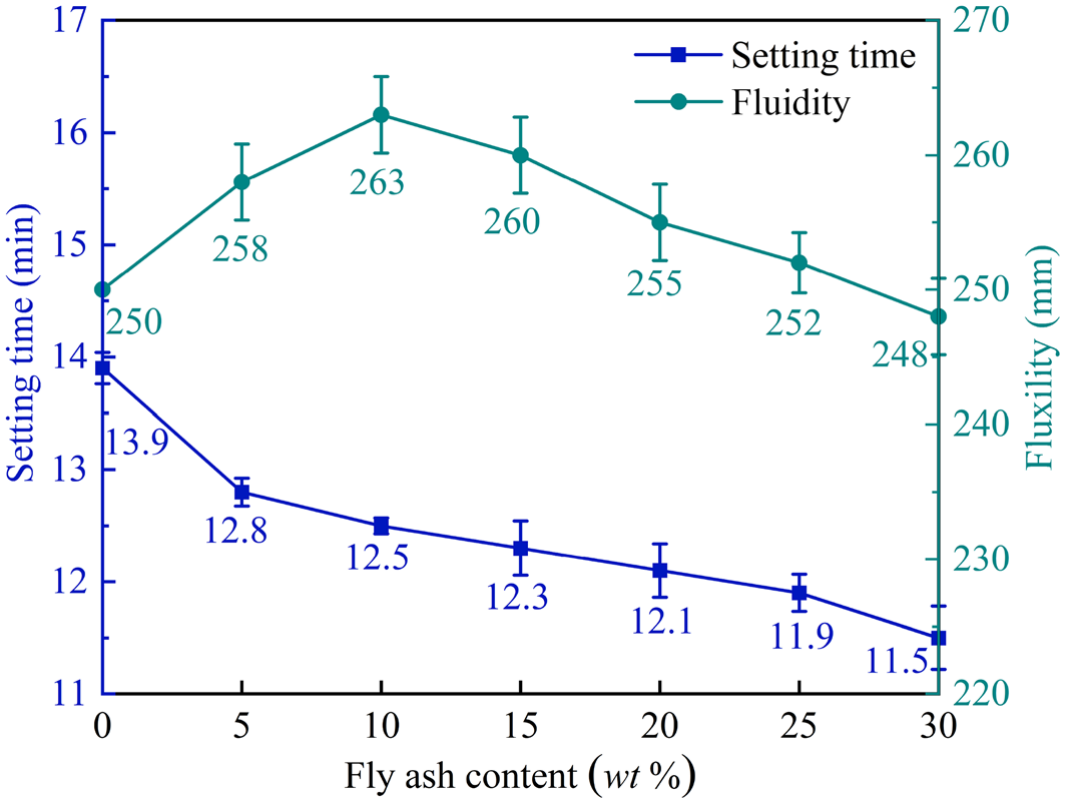
Setting time and fluidity of MPM with different fly ash content.

It can be seen from Fig. 5 that the setting time of MPM cured at 20 °C decreases with the increasing of fly ash content. On the one hand, fly ash partially replaces NH_4_H_2_PO_4_ and MgO, which reduces the concentration of MgO and the dosage of retarder in MPM system, and delays the dissolution of MgO and acid–base interaction. On the other hand, fly ash has higher water absorption, the increasing of fly ash content results in a reduction of free water involved in hydration reaction and then shortens the setting time of MPM.

The fluidity of MPM slurry increases first and then decreases with the increasing of fly ash content. When with lower fly ash content, the spherical fly ash particles play a ball lubrication role and thereby improve the fluidity of MPM slurry^24^. When the fly ash content exceeds 10 wt%, the excessive fine fly ash particles expand the total specific surface area of raw materials of MPM, the surface area that required water infiltration increases accordingly, which causing a decrease in fluidity.

#### Mechanical properties of MPM with different fly ash content

**Figure 6:**
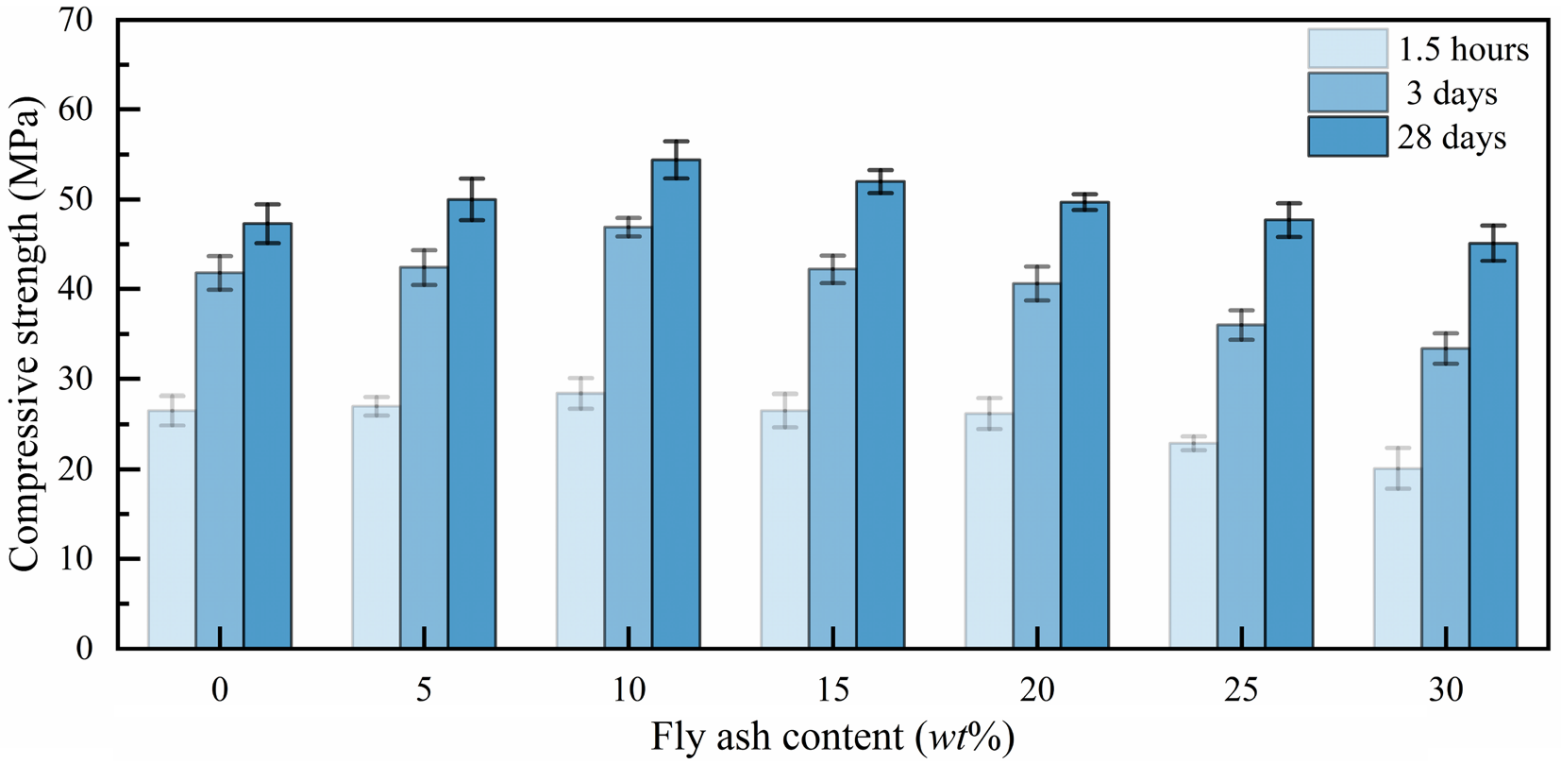
Compressive strength of MPM with different fly ash content.

**Figure 7:**
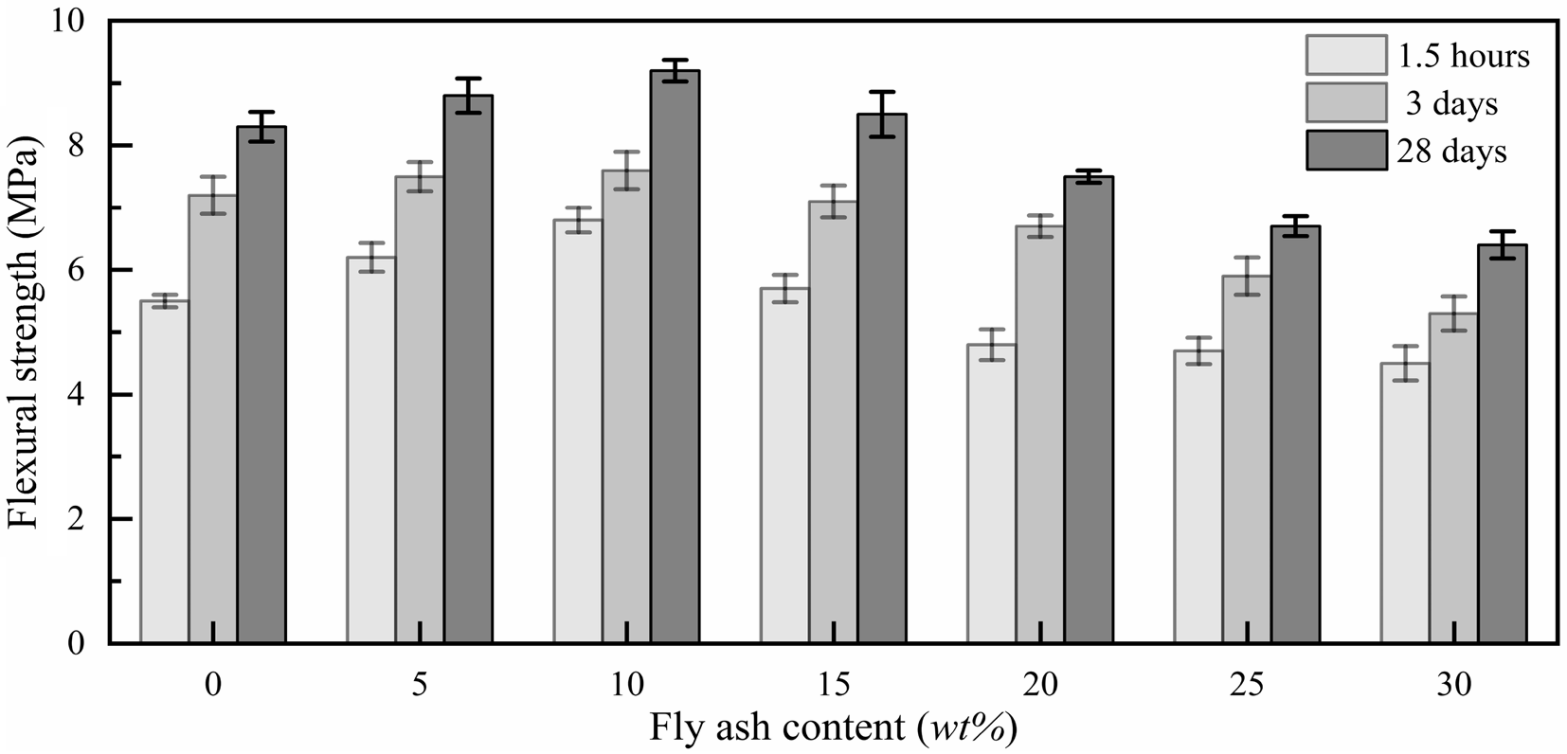
Flexural strength of MPM with different fly ash content.

It can be seen from Figs. 6 and 7 that the compressive strength and flexural strength of MPM at each curing age increase first and then decrease with the increasing of fly ash content, and both reach the best when the fly ash content is 10 wt%. Compressive strength and flexural strength of MPM-10 cured for 28 days increases 12.2% and 10.8% compared with MPM-0 respectively. Since the particle size of fly ash is smaller than that of MgO, it acts as a crystal nucleus, which continuously promotes the formation of hydration products and fills the pores of the hardened structure, and thereby improves the mechanical properties of MPM^17^. Because the reactivity of fly ash is lower than that of MgO in acidic environment, when the content of fly ash is too high, the bonding performance of hardened MPC decreases, resulting in the decrease of mechanical properties of MPM^25^.

#### Interfacial bonding strength of MPM with different fly ash content

**Figure 8:**
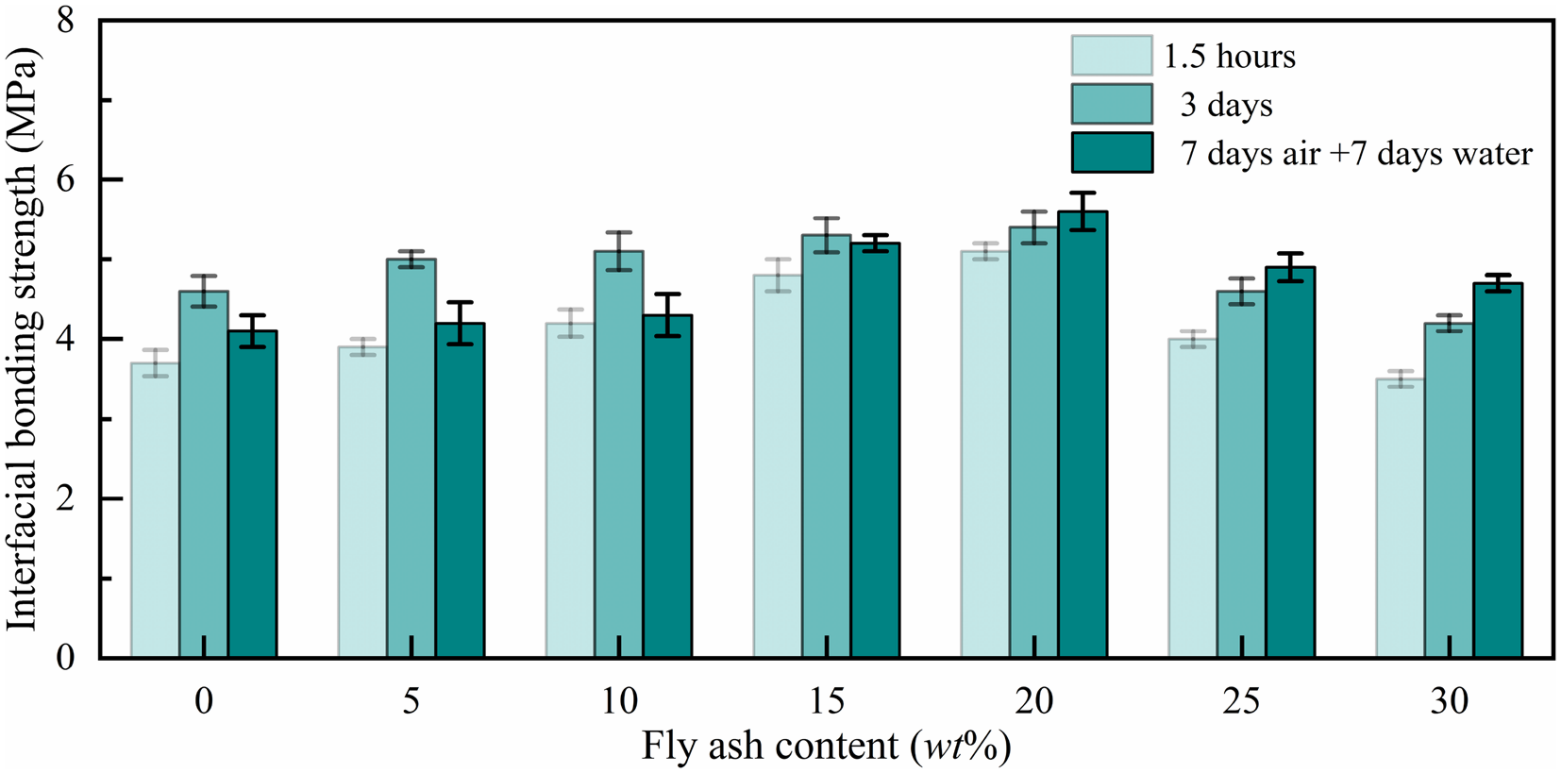
Interfacial bonding strength of MPM with different fly ash content.

Figure 8 shows the interfacial bonding strength of MPM curing at 20 °C, which increases first and then decreases with the increasing of fly ash content, and reaches the best when the fly ash content is 20 wt%. Interfacial bonding strength of MPM-20 cured for 3 days reaches 5.4 MPa, increasing 17.4% compared with MPM-0. At lower fly ash content, the bonding performance of MPM is higher during the early hydration stage of MPM. In the repair interface area between the Portland cement mortar and MPM, Ca(OH)_2_ and fly ash take place the secondary hydration reaction and generates C–S–H gel, which improves the bonding performance of MPM, so the interfacial bonding strength is slightly promoted. When the content of fly ash is more than 20 wt%, the cohesive struvite in the hardened structure decreases, and the interfacial bonding strength declines accordingly. Moreover, the amount of Ca(OH)_2_ enriched in interface area is finite, and the secondary hydration is also limited.

It should be noted that the interfacial bonding strength of MPM specimens, which were air cured 7 days followed by water curing 7 days, is slightly higher than that of air cured for 1.5 h and for 3 days when the fly ash content is not less than 20 wt%. The infiltration of water during immersing accelerates the leaching and migration of Ca(OH)_2_ in the interface area between Portland cement mortar and MPM, stimulates the pozzolanic activity of unreacted fly ash, and increasing interfacial bonding strength of MPM^26^.

#### Water resistance of MPM with different fly ash content

**Figure 9:**
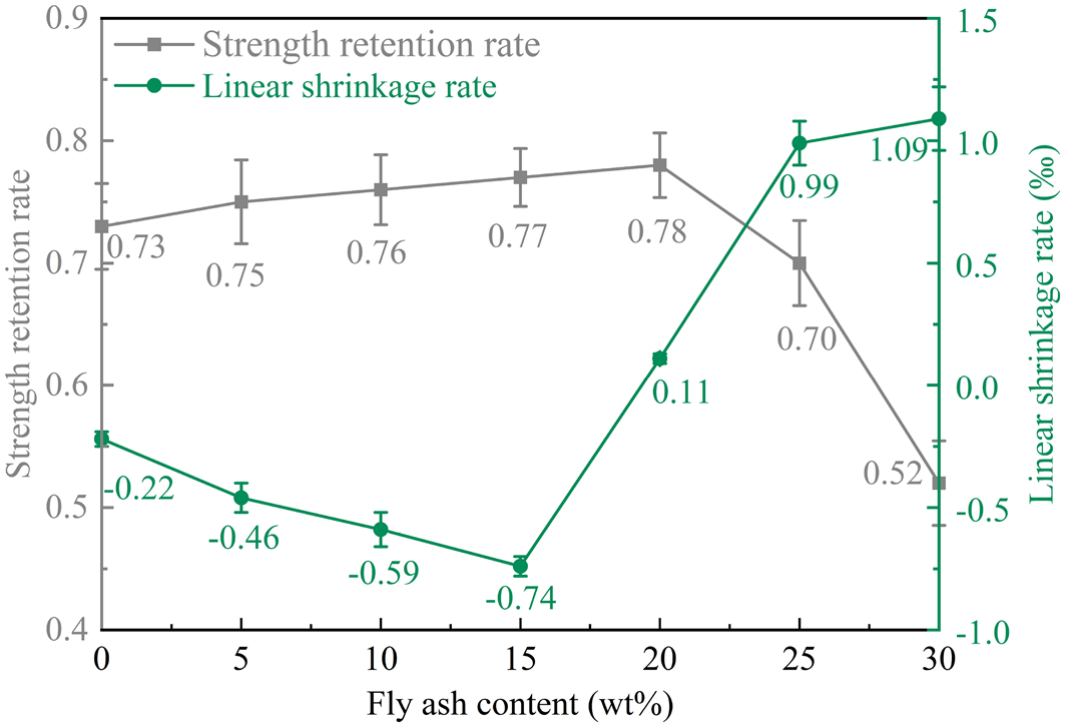
Water resistance and dry shrinkage rate of MPM with different fly ash content.

From Fig. 9, it can be seen that the strength retention rate of MPM cured at 20 °C increases from 0.73 to 0.78 when the fly ash content rises to 20 wt%, and decreases significantly as it continues to increase. Because activated silica and alumina enriched in fly ash reacts with soluble phosphates in the hydration products of MPM^27^. Meanwhile, the finer fly ash particles improve water stability by refining the pore structure, making the microstructure denser. The combined effect of two preceding actions helps to alleviate MPM from water corrosion. The more fly ash is mixed, the less MgO and NH_4_H_2_PO_4_ are required, resulting in the reduction in struvite, the compressive strength and the strength retention rate are decreased accordingly.

#### Linear shrinkage rate of MPM with different fly ash content

Figure 9 shows that the linear shrinkage rate of MPM is negative when fly ash content is not higher than 15 wt%, which means that MPM is slightly expanded during drying, and decreases with fly ash content increasing. Thereafter, the linear shrinkage rate of MPM increases with the fly ash content, all of which are positive, that is, occurring a slight shrinkage. Comparing with MPM-0, MPM-15 shows a 0.52‰ reduction in line shrinkage, while MPM-30 increasing 1.31‰. The activated aluminum silicate contained in fly ash reacts with phosphate and generate hydration products with cementitious properties, which was filled in the pores and caused a slight expansion of MPM^28^.

As the fly ash content gradually increasing to 15 wt%, the linear shrinkage rate increases rapidly. This is because the increasing in fly ash dosage reduces the aggregation of struvite in hydration products of MPM^29^. Furthermore, most of the water around fly ash particles does not participate in the hydration reaction, with the formation of ammonia and water during MPM hydration, which is help to form connected pore. During drying process, the reduction in struvite and its continuity and increase in connected pores accelerate moisture dissipation from MPM, leading to an increment of linear shrinkage.

#### Micro-morphology and mineral composition of MPM with different fly ash content

**Figure 10:**
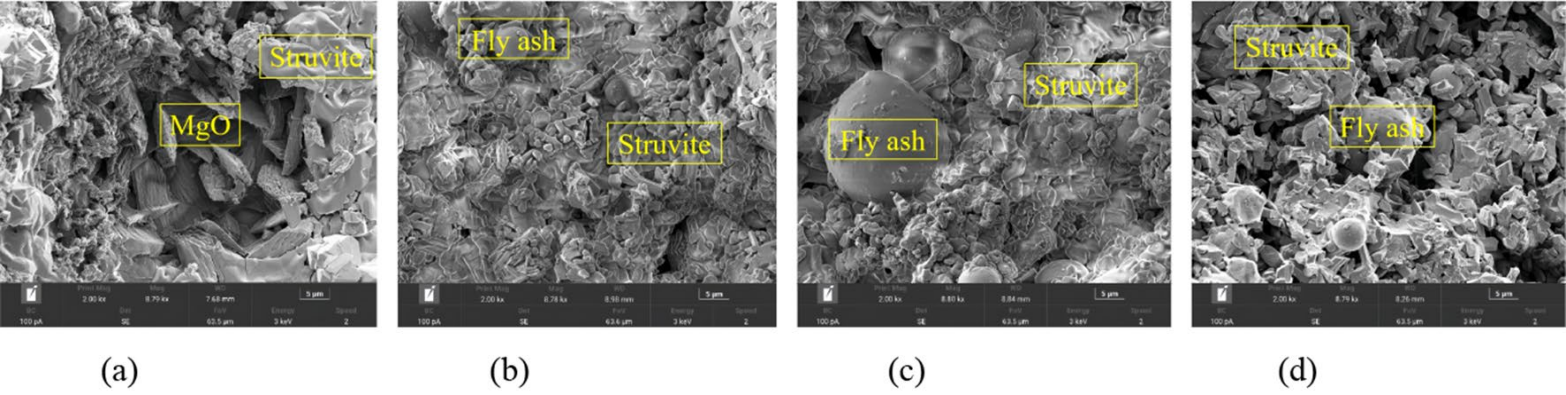
SEM images of MPM cured at 20 °C for 28 days. (**a**) MPM-0, (**b**) MPM-10, (**c**) MPM-20, (**d**) MPM-30.

Figure 10 shows the micro-morphology of MPM with different fly ash content hydrated at 20 °C for 28 days. Struvite and dead-burned MgO crystals can be seen in Fig. 10a, and the former is comparatively continuous. In Fig. 10b, fly ash particles, struvite and MgO can be observed, and the refined struvite adheres to the surface of fly ash particles, which slightly increases the compactness of hardened MPM. It can be seen from Fig. 10c and d that the crystallinity and continuity of struvite further decrease comparing to MPM-10, and the compactness of the hardened structure also reduces. The SEM images suggest that too much incorporation of fly ash reduces the generation of struvite and decreases the densification of the hardened MPM, thereby deteriorating its mechanical properties and water resistance.

**Figure 11:**
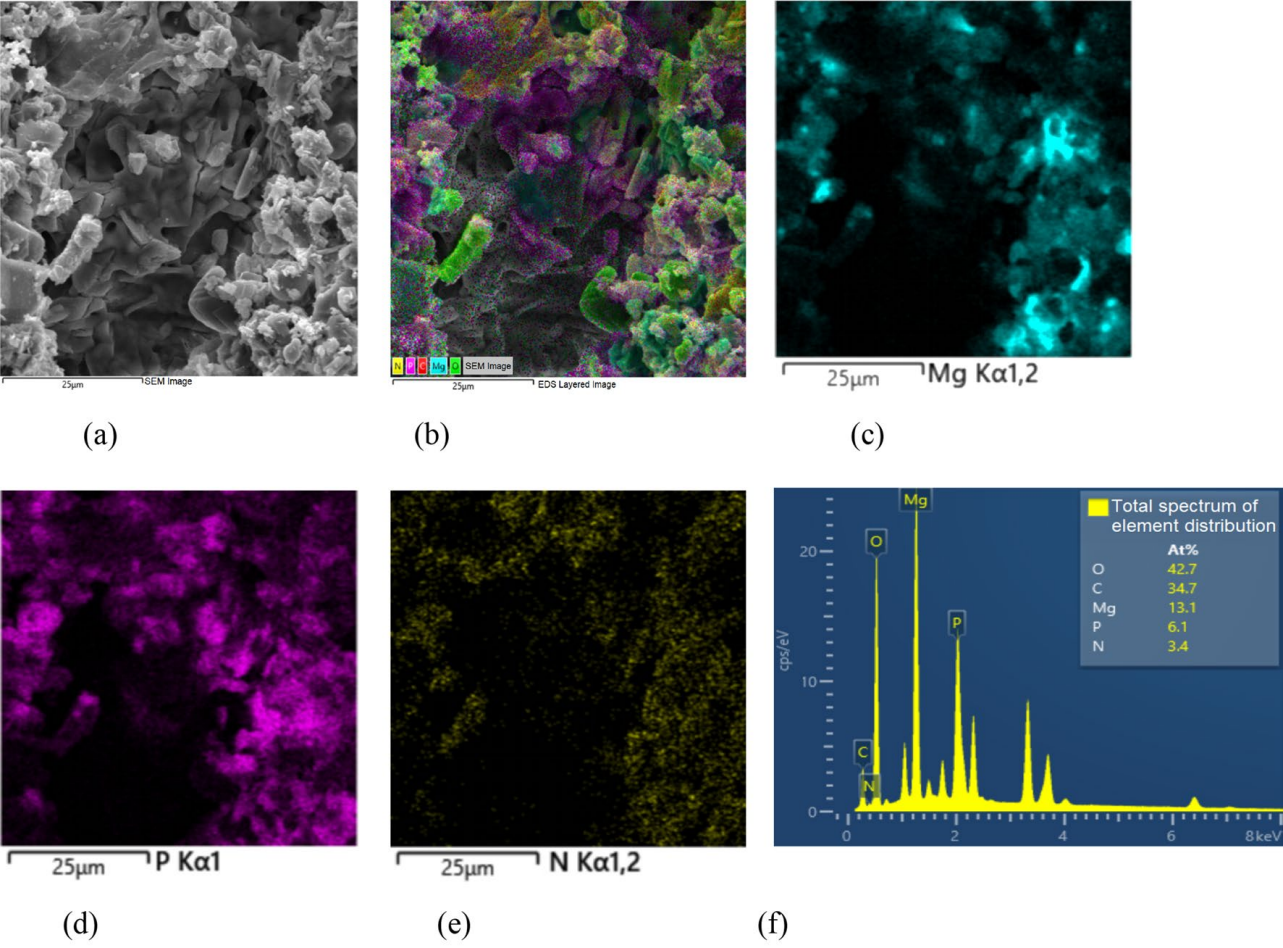
EDS analysis of MPM-10 cured at 20 °C for 28 days. (**a**) SEM images, (**b**) EDS layered images, (**c**) Mg element Kα, (**d**) P element Kα, (**e**) N element Kα, (**f**) Total spectrum of element distribution.

It can be seen from Fig. 11a that the hydration structure of MPM-10 is dense, and struvite and fly ash particles are also be clearly observed. The EDS layered scanning image in Fig. 11b shows that the hydration products of MPM-10 are mainly composed of Mg, P, N and O. Figure 11c–e display the Kα highlight reflections from elements of Mg, P and N, respectively, which are uniformly distributed around and on the surface of spherical fly ash particles. Because of adopting surface layer scanning, bright reflections are not shown in areas far from the inner layer. Figure 11f shows the total spectrum of element distribution in the hydration products of MPM-10, indicating that MgO was obviously excessive after hydration reaction, which is consistent with the research results of relevant literature^2,6^.

**Figure 12:**
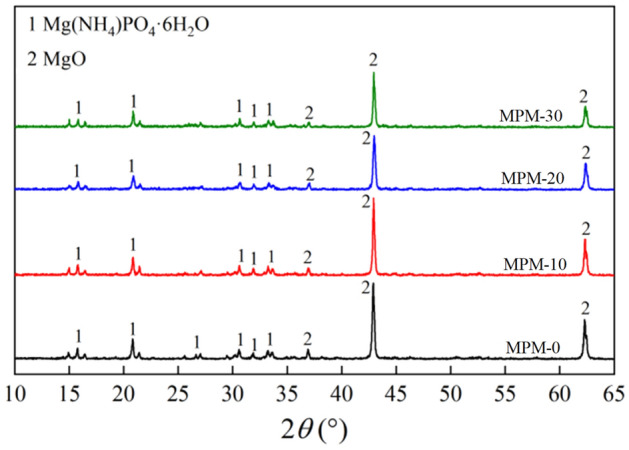
XRD spectrum analysis of MPM cured at 20 °C for 28 days.

Figure 12 exhibits the hydration XRD spectrum with various fly ash contents cured at 20 °C for 28 days. It is evident that Mg(NH_4_)PO_4_·6H_2_O (struvite) and dead-burned MgO are the two primary phase groups of hardened MPM net slurry. The results in Fig. 12 is in agreement with those in Fig. 11. With the increase of fly ash content, the diffraction intensity of the characteristic peaks gradually decreases, and there is no obvious characteristic peak of mineral composition in the XRD pattern because the fly ash particles are wrapped or isolated by the dead-burned MgO and hydration products, as can be observed in Fig. 10.

As can be seen from Figs. 10, 11 and 12, the hydration products of MPM are not significantly changed by the incorporation of fly ash, but their densities are somewhat increased with fly ash dosage. When the replacement rate of fly ash is 20 wt%, the densification of the hydrated structure is at its highest, and fly ash particles are sufficiently enveloped by struvite. When replacement rate continues to increase, the hydration structure gets looser, and the degree of retention of fly ash particles decreases, which leading to a corresponding decrease in water resistance and mechanical properties of MPM.

### Physical and mechanical properties of MPM-10 at different curing temperatures

From above results, when the fly ash content is 10 wt%, the mechanical properties and water resistance of MPM are all optimized in comparison to MPM-0. Therefore, MPM-10 was taken as a reference group to clarify the effect of curing temperature on its performance in the following.

#### Setting time and fluidity of MPM-10 which raw materials pretreated at different temperatures

**Figure 13:**
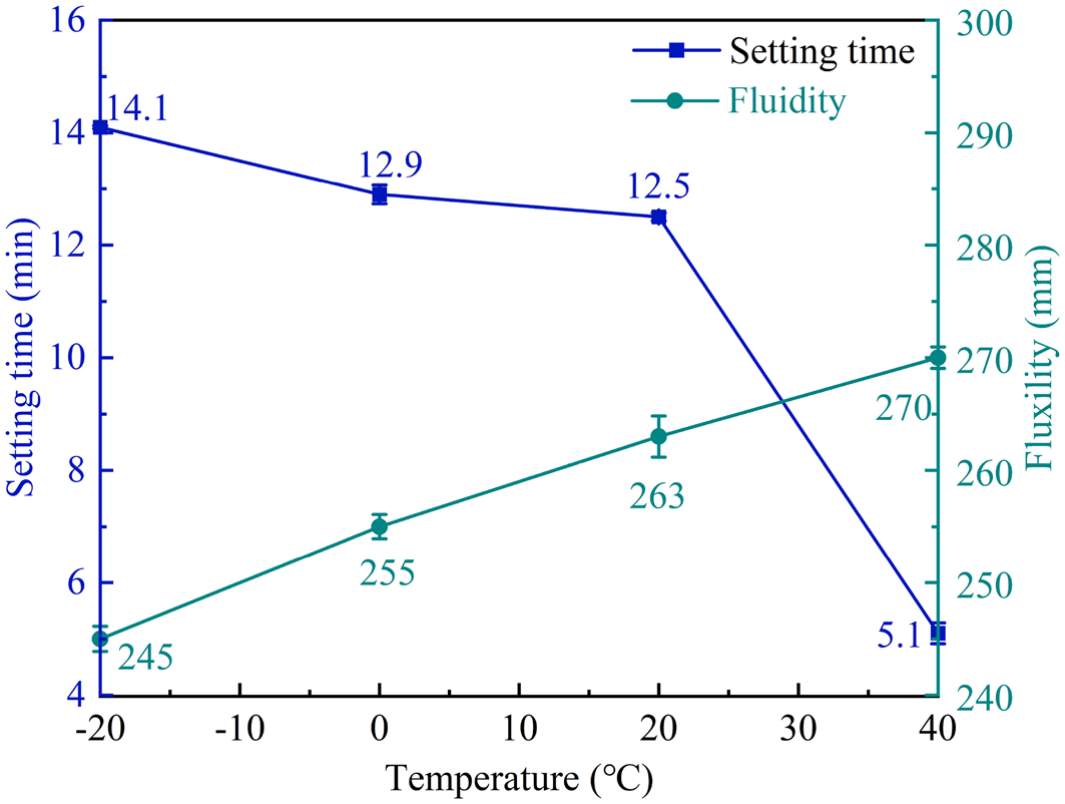
Setting time and fluidity of MPM-10 at different pretreated temperatures.

As can be seen from Fig. 13 that the setting time of MPM-10 decreases with the increasing in the pretreatment temperature of its raw materials. When the pretreatment temperature reaches 40 °C, the setting time is only 5.1 min. As the pretreated temperature raises from 0 to 40 °C, the solubility of NH_4_H_2_PO_4_ increases significantly, and it is 0.567 g/ml at 40 °C, which is over 2.5 times higher than that at 0 °C. As a result, the acidity of fresh mixture elevates with the pretreatment temperature, the dissociation rate of dead-burned MgO increases correspondingly, accelerating the hydration reaction rate of MPM-10^30^.

The fluidity of MPM-10 increases slightly with the increasing of pretreating temperature. At the same ambient temperature, the temperature of fresh mixture increases with the ascending in raw material temperature, the viscosity of NH_4_H_2_PO_4_ solution decreased correspondingly, and then increasing in the fluidity of fresh mixture.

#### Mechanical properties of MPM-10 cured at different temperatures

**Figure 14:**
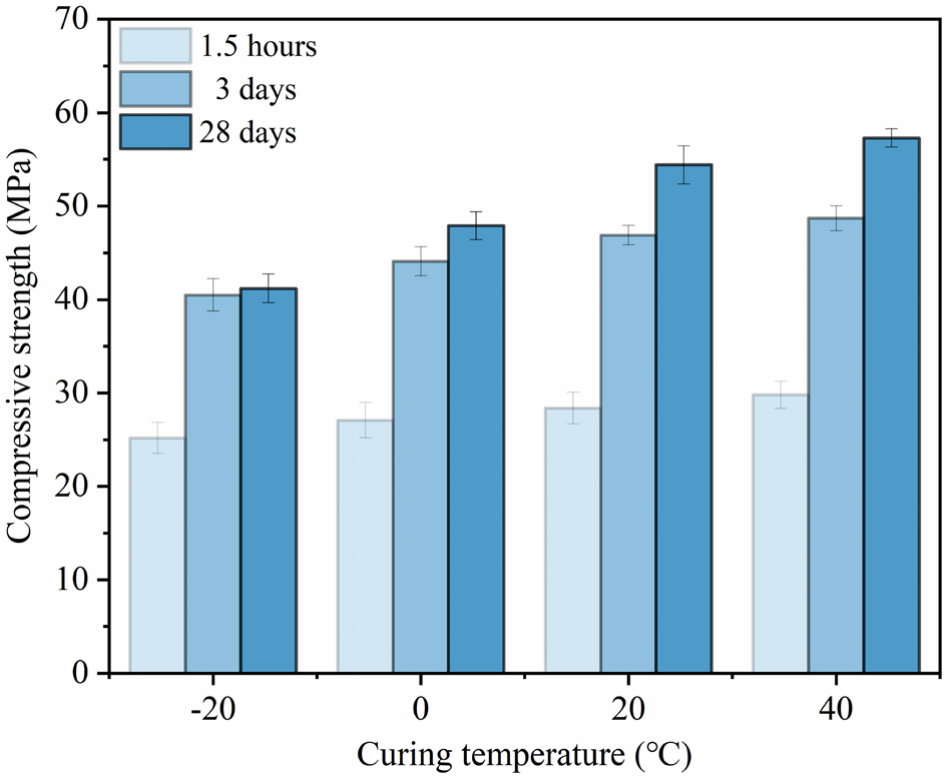
Compressive strength of MPM-10 at different curing temperatures.

From Fig. 14, it can be seen that the compressive strength of MPM-10 cured for various ages increases with the elevating in curing temperature. Comparing with the specimens cured at 20 °C for 1.5 h and 28 days, the compressive strength of MPM-10 cured at 0 °C reduces 6.5% and 11.9% respectively, while MPM-10 cured at − 20 °C, its decreases are 11.3% and 24.3%. The results suggest that the cold environment has a significant effect on the later performance of MPM-10, and the lower curing temperatures, the increase is the more prominent.

On the one hand, the negative temperature inhibits the hydration reaction rate of MPM-10, and the decrease in temperature also reduces the participation of raw materials in the acid–base reaction and activity of fly ash, which leads to a decrease in the generation of hydration products. Therefore, the compressive strength of MPM-10 cured at negative temperature reduces comparing with the specimens cured at positive temperature. On the other hand, the elevating of curing temperature improves the reactivity of fly ash, and its secondary hydration reaction enhances the filling effect. In addition, accelerated dissolution NH_4_H_2_PO_4_ and MgO elevates hydration temperature and reaction rate, which causing an increase in struvite generation, and then compressive strength of MPM-10 increases.

**Figure 15:**
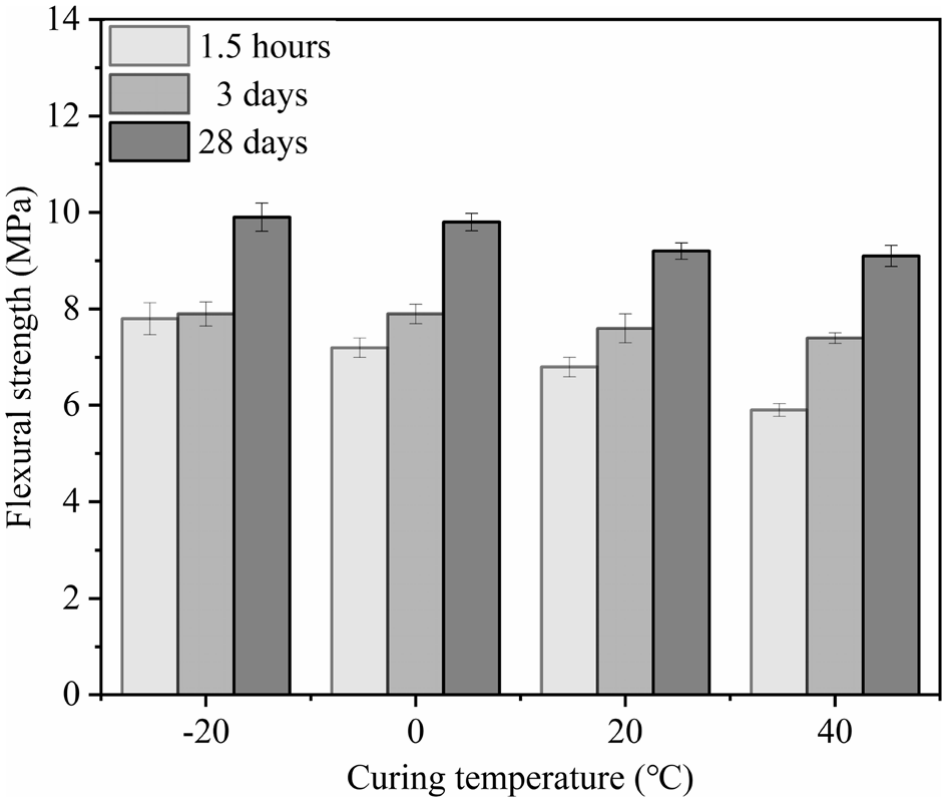
Flexural strength of MPM-10 at different curing temperatures.

As can be seen from Fig. 15 that the flexural strength of MPM-10 decreases slightly with the elevating in curing temperature. When the curing temperature is − 20 ℃, the flexural strength of MPM-10 reaches 9.9 MPa for the 28 days specimens. Under lower temperature curing, hydration reaction rate of MPM-10 decreases at the initial stage. The remaining dead-burned MgO increases with declining curing temperature after hardening, which contributes to improve the flexural strength of MPM-10. At the same time, with the decreasing of curing temperature, the viscosity of pore solution and the mobility of capillary in hardened MPM-10 decreases, and its resistance to bending load increases accordingly^31^. In addition, when the curing temperature is − 20 °C, the moisture in pore solution exists in ice, which also helps to improve flexural strength.

#### Interfacial bonding strength of MPM-10 cured at different temperatures

**Figure 16:**
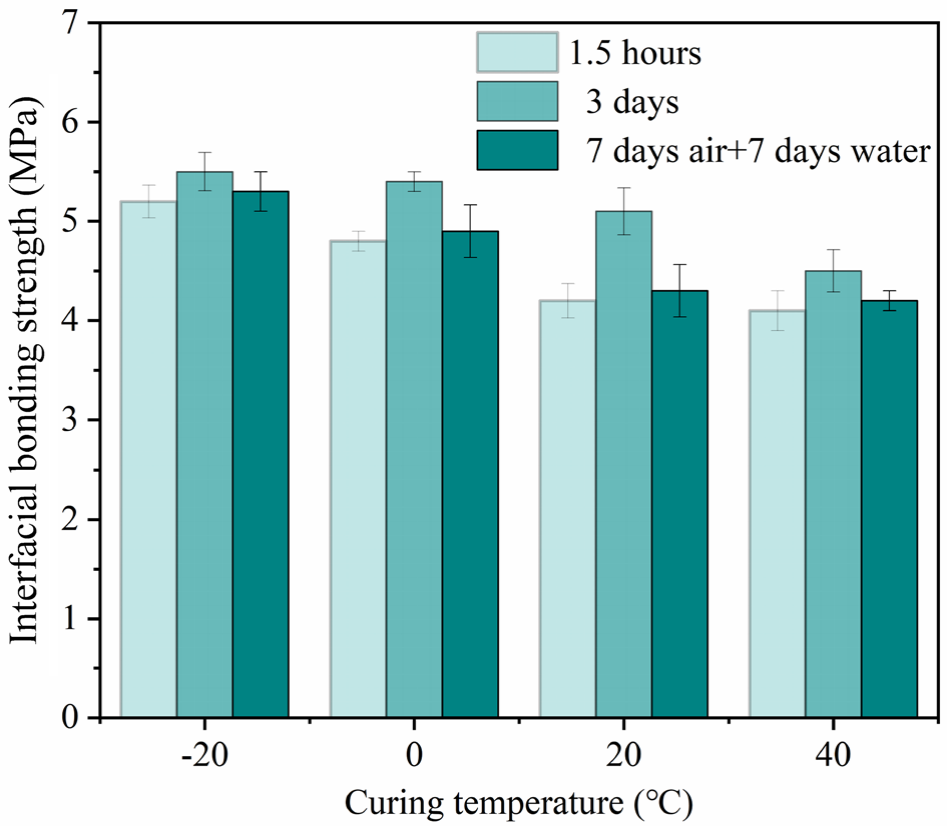
Interfacial bonding strength of MPM-10 at different curing temperatures.

As can be seen from Fig. 16 that the interfacial bonding strength of MPM-10 at different ages decreases with the elevating in curing temperature, which is consistent with the change trend of its flexural strength. When the curing temperature is − 20 ℃, the interfacial bonding strength of MPM-10 is the highest, reaching 5.5 MPa for the cured 3 days specimens. The bonding strength between the repair materials and the cement mortar is a comprehensive result of physical and chemical adhesion, the former is determined by the adhesive ability of the repair mortar itself, and the latter depends on ion transmission between bonding surfaces^32^. For MPM, the chemical bonding force comes from the reaction between phosphate in the hydration product and Ca(OH)_2_ enriched in the interface region of ordinary mortar. With the ascending of curing temperature, the setting time of MPM-10 was significantly shortened (as seen in Fig. 13), and the ion transporting time between bonding surfaces of slurry and ordinary mortar is correspondingly shortened, which reduces its interfacial bonding strength.

#### Water resistance of MPM-10 cured at different temperatures

**Figure 17:**
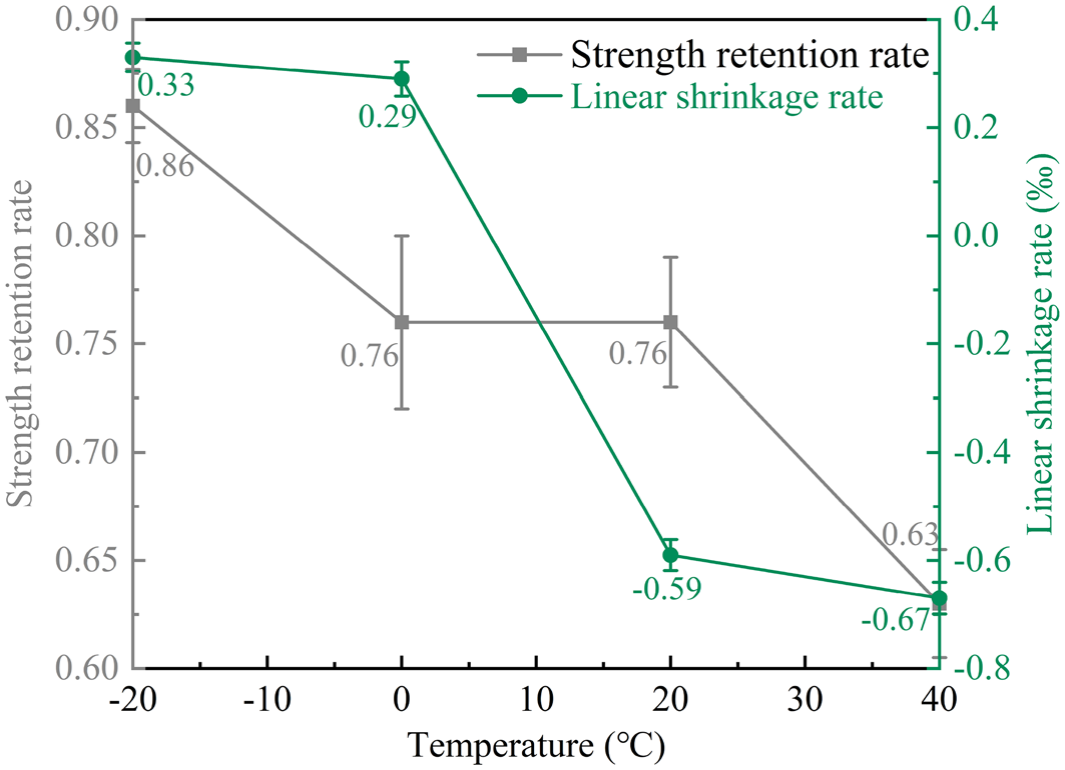
Strength retention and linear shrinkage rate of MPM-10 at different curing temperatures.

The results of Fig. 17 show that the strength retention rate of MPM-10 decreases with the elevating in curing temperature. When MPM-10 is cured at 40 °C, strength retention rate is 0.63, which is 26.7%, 17.1%, and 17.1% lower than that of specimens cured at − 20 °C, 0 °C, and 20 °C, respectively. Contrarily, the strength retention of MPM-10 cured at − 20 °C is 13.2% higher than that of 20 °C cured specimen. MPM hydrates quickly and reaches final setting in a very short time, while releasing a large amount of hydration heat and ammonia. When curing at higher temperatures, rapid solidification prevents ammonia from escaping, which leads to a decrease in the compactness of hardened MPM-10 and thus its water resistance.

#### Linear shrinkage rate of MPM-10

As can be seen from Fig. 17 that the linear shrinkage rate of MPM-10 decreases with the increasing of curing temperature. When the curing temperature is − 20 °C and 0 °C, MPM-10 inhabits linear shrinkage during hardening, while it is 20 °C and 40 °C, MPM-10 occurs linear expansion. When the raw materials are pretreated at − 20 °C and 0 °C, the struvite generation and density of hardened MPM-10 before demolding are significantly lower than those of the specimens pretreated at 20 °C and 40 °C. The linear shrinkage test specimens were immersed immediately in liquid water after demolding and water cured for 3 days at different curing temperatures. During this period, the water absorption of the MPM-10 is significantly affected by the initial pore structure. The more water is absorbed, the more water is lost during subsequent air curing, and the greater linear shrinkage is. In addition, when MPM-10 is air cured at − 20 °C and 0 °C, where relative humidity is close to 0, and almost all of the absorbed moisture is dissipated during linear shrinkage test, so their linear shrinkage is larger than that of specimens cured at 20 °C and 40 °C.

## Conclusion

In this paper, the influence mechanism of fly ash content on the physical and mechanical properties and microstructure of magnesium phosphate repair mortar (MPM) was studied. The properties of fly ash modified MPM cured at various temperature were further explored, focusing on the effect of negative temperature environments. The following conclusions are drawn from above studies:

1. The physical and mechanical properties of MPM are slightly improved in comparison to the original when fly ash content is 10 wt% and 15 wt%. The setting time of MPM decreases with the increasing of fly ash content. The fluidity, mechanical properties, interfacial bonding strength and water resistance increase first and then decrease with fly ash content, among them, the compressive strength and flexural strength reach the best when the fly ash content is 10 wt%, and the interfacial bonding strength and strength retention rate reach the best when the fly ash content is 20 wt%. The linear shrinkage rate of MPM decreases first and then increases with the increasing of fly ash content, it is the lowest when fly ash content is 15 wt%, and is − 0.74‰.
2. The participation of fly ash reduces the continuity of the hydration products of MPM, accompanying with a little increase in its densification. The hydration products of MPM are mainly struvite and dead-burned MgO crystals. Struvite crystals of the original MPM are integrity. For the fly ash modified MPM, crystallinity and continuity of struvite obviously decrease, and fly ash particles and its fibrous hydration products also can be observed. Mg, P and N elements are evenly distributed in the hardened MPM modified by 10 wt% fly ash, and fly ash particles are surrounded with fine struvite and unreacted MgO.
3. MPM-10 still characterizes with early strength, rapid hardening, good water resistance and interfacial adhesion in environments ranging from − 20 to 0 ℃. With the increase of raw material pretreating temperature, the setting time of MPM-10 becomes shorter and the fluidity increases linearly. The compressive strength of MPM-10 cured at various ages shows an increase with the raising in curing temperature, while the flexural strength and interfacial bonding strength show an opposite trend. When the curing temperature is − 20 ℃, the interfacial bonding strength and flexural strength of MPM-10 are 5.5 MPa and 7.9 MPa for the cured 3 days specimens, respectively. The strength retention rate and linear shrinkage rate of MPM-10 decrease with the increase in curing temperature.

## Future work

Future work will focus on the electrical properties and their intelligent sensibility of carbon fiber modified magnesium phosphate repair mortar at severe cold environment.

## Data Availability

The datasets generated during and/or analysed during the current study are available from the corresponding author on reasonable request.
